# Multi-level magma plumbing at Agung and Batur volcanoes increases risk of hazardous eruptions

**DOI:** 10.1038/s41598-018-28125-2

**Published:** 2018-07-12

**Authors:** Harri Geiger, Valentin R. Troll, Ester M. Jolis, Frances M. Deegan, Chris Harris, David R. Hilton, Carmela Freda

**Affiliations:** 1 Section for Mineralogy, Petrology and Tectonics, Department of Earth Sciences, Uppsala University, Uppsala, Sweden; 2 Istituto Nazionale di Geofisica e Vulcanologia, Rome, Italy; 3 Faculty of Geological Engineering, Universitas Padjajaran (UNPAD), Bandung, Indonesia; 4 Geomar Helmholtz Centre for Ocean Research, Kiel, Germany; 5 Department of Geological Sciences, University of Cape Town, Cape Town, South Africa; 6 Geosciences Research Division, Scripps Institution of Oceanography, La Jolla, USA

## Abstract

The island of Bali in Indonesia is home to two active stratovolcanoes, Agung and Batur, but relatively little is known of their underlying magma plumbing systems. Here we define magma storage depths and isotopic evolution of the 1963 and 1974 eruptions using mineral-melt equilibrium thermobarometry and oxygen and helium isotopes in mineral separates. Olivine crystallised from a primitive magma and has average δ^18^O values of 4.8‰. Clinopyroxene records magma storage at the crust-mantle boundary, and displays mantle-like isotope values for Helium (8.62 R_A_) and δ^18^O (5.0–5.8‰). Plagioclase reveals crystallisation in upper crustal storage reservoirs and shows δ^18^O values of 5.5–6.4‰. Our new thermobarometry and isotope data thus corroborate earlier seismic and InSAR studies that inferred upper crustal magma storage in the region. This type of multi-level plumbing architecture could drive replenishing magma to rapid volatile saturation, thus increasing the likelihood of explosive eruptions and the consequent hazard potential for the population of Bali.

## Introduction

Volcanic eruptions and their products are not only hazardous to populations and infrastructure in their direct vicinity, but large eruptions can also affect global climate and thus society as a whole^1,2^. Even relatively minor eruptions can have disastrous socio-economic impacts, such as the eruption of Eyjafjallajökull on Iceland in 2010, which caused major air traffic disruptions over large parts of northern Europe^3^. To be better prepared for volcanic events and their repercussions, an understanding of the inner workings of active volcanoes and their underlying plumbing systems is of utmost importance^4^.

Indonesia is one of the world’s most densely inhabited nations and has a rapidly growing population and economy^5^. Bali Island has, moreover, a high transient population with seasonal peaks due to its large tourism sector and it is home to two active volcanoes, Agung and Batur. At the time of writing, Agung was in a state of unrest^6^, but prior to that, the last major eruption was in 1963 following 120 years of dormancy^7^. The 1963 eruption destroyed large areas on the volcano’s flanks and caused ~2000 fatalities, which makes it the most devastating eruption in Indonesia since the 1883 eruption of Krakatau and one of the most significant volcanic events of the 20^th^ century^2,7^. Batur erupted 27 times since 1804, with the most recent eruptions in 1994, 1998, and 1999–2000 (ref.^8^). Most notably, Batur erupted contemporaneously with Agung in 1963 (ref.^9^), but Agung did not erupt simultaneously with Batur in 1974.

Here we employ petrological and geochemical approaches (mineral compositional data, oxygen isotopes in olivine, clinopyroxene and plagioclase, helium isotopes in clinopyroxene, and mineral-melt equilibrium thermobarometry) on lavas from the 1963 eruption of Agung and the 1963 and 1974 eruptions of Batur to better constrain the supply systems that feed these two volcanoes and to ultimately help improve eruption forecasting. We then consider the results of our study in conjunction with available thermobarometric and oxygen isotope data from other Sunda arc volcanoes in an attempt to identify arc-wide patterns in magma storage and associated crustal magma differentiation.

## Geological Background

**Figure 1:**
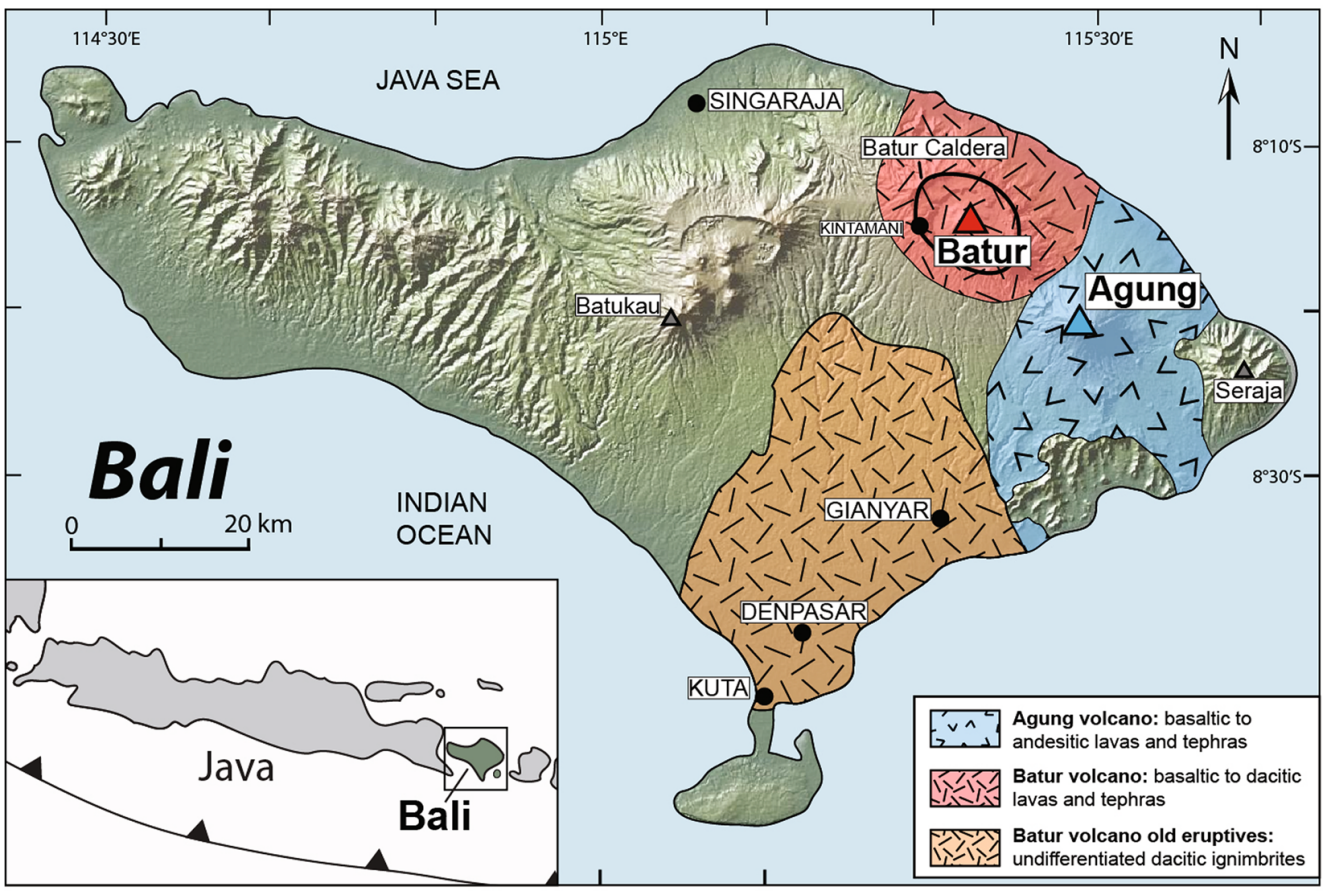
Location map for Agung and Batur volcanoes. Simplified geological map of Bali, Indonesia (modified after Reubi and Nicholls)^13^ overlain onto a DEM (Source: GeoMapApp, http://www.geomapapp.org)^71^. The locations of Agung (blue) and Batur (red) volcanoes are marked, as is the extent of their erupted products. The inset shows the location of Bali within the Sunda Arc.

Agung and Batur are active Sunda arc stratovolcanoes located on the island of Bali (Fig. 1). The Sunda arc subduction system spans over 5600 km from the Andaman Islands in the west to Banda Island in the east^10–12^. Bali belongs to the Lesser Sunda Islands located east of Java in the central part of the Sunda arc segment^13^, which formed as a result of northward subduction of the Indo-Australian plate beneath the Eurasian plate at ~6 to 7 cm/year^10,12,14,15^. The MOHO underneath Bali is located at ~18–20 km depth and the crust displays an oceanic velocity structure^16^. The central and northern part of the island hosts four Quaternary volcanic fields (Fig. 1; Batukau, Bratan Caldera, Batur Caldera and Agung). Of these, Batur and Agung have been the only active volcanoes in historical times^13,17^.

Agung volcano (8°25′S, 115°30′E; 3142 m asl) dominates the eastern part of Bali (Fig. 1) and besides eruptions in 1843 and 1963, intense solfatoric activity was observed in 1908, 1915, and in 1917. The 1963 eruption emitted 0.95 km³ dense rock equivalent (DRE) of basaltic andesite and andesite tephra and lava and attained a maximum eruptive column height of 28 km (ref.^9^). Moreover, it is estimated that the 1963 eruption released between 1.9 and 3.4 Mt of Cl and between 7 and 7.5 Mt of SO_2_^9,18^. The amount of released climate-active gases caused a subsequent drop of 0.3 °C in average northern hemisphere temperatures, making this atmospheric perturbation the fourth largest of its kind in the 20^th^ century^9^.

Batur volcano (8°14′S, 115°22′E; 1717 m asl) is located to the north-west of Agung and in the direct vicinity of lake Danau Batur^13,17^ (Fig. 1). The Batur stratovolcano is located within the Batur Volcanic Field (BVF), a double caldera structure that formed through two successive collapses at ~29,300 and ~20,150 years BP and which produced over 100 km³ of dacitic ignimbrite^12,13^. These two caldera-forming eruptions were followed by several smaller events that erupted a broader compositional range from basalt to rhyolite, although the most recent erupted products are restricted to basaltic andesite compositions^13,19,20^.

## Results

### Mineral Chemistry and Petrography

#### Agung

**Figure 2:**
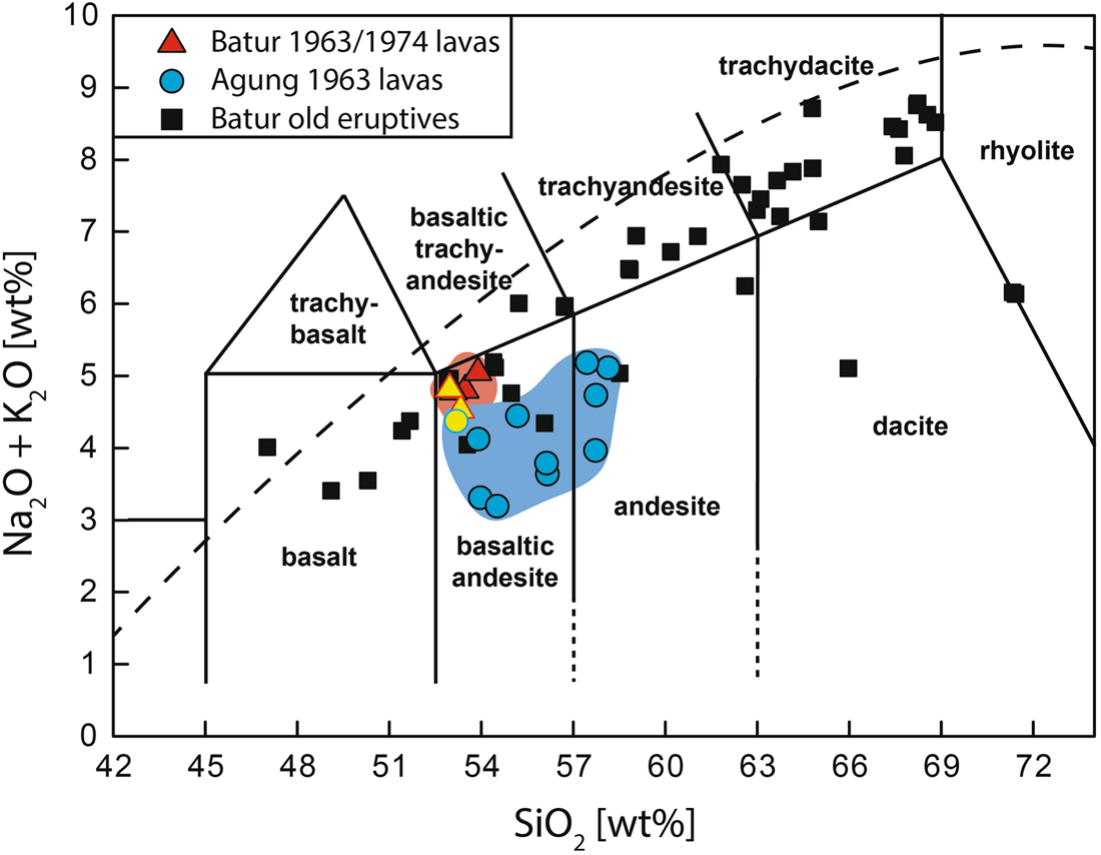
Total alkali versus silica (TAS) diagram for Agung and Batur eruptives. Lavas from the 1963 eruption of Agung are basaltic andesite and andesite. Lavas from the 1963 and 1974 eruptions of Batur also plot in the basaltic-andesite field. In contrast, Batur post-caldera lavas plot in the basalt field whereas older eruptives of Batur span from basalt to rhyolite. Data from this study are marked by yellow symbols. Data were normalized on a volatile-free basis. Additional data sources^9,12,15,17,19,20^.

**Figure 3:**
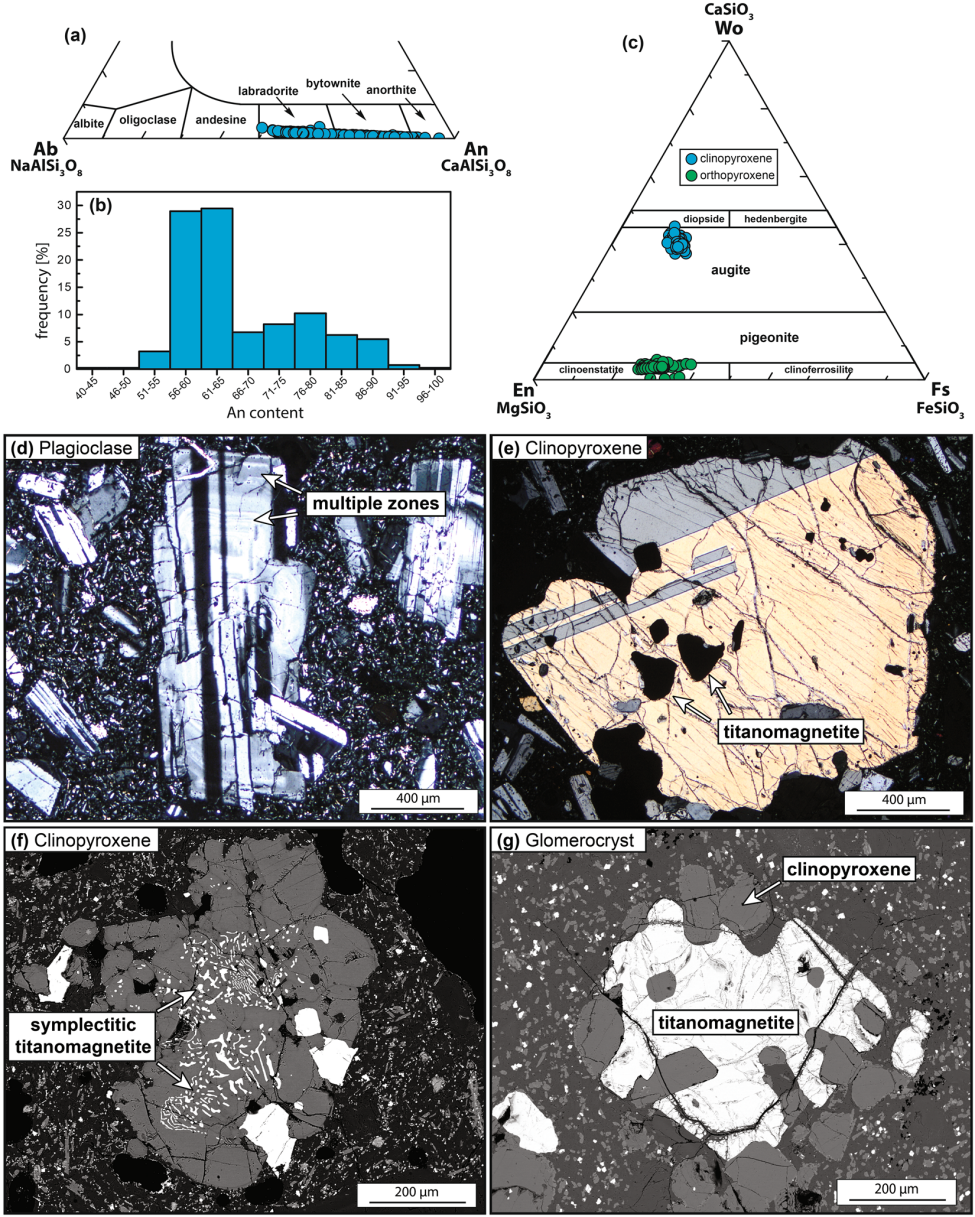
Mineral chemistry and petrography for Agung. (**a**) Compositions of plagioclase from the 1963 Agung eruption (n = 401), which classify as labdradorite, bytownite and anorthite. (**b**) Frequency of anorthite content in Agung plagioclase in the range of An_42–96_. (**c**) Compositions of clinopyroxene and orthopyroxene from the 1963 eruption of Agung. Clinopyroxene classify as diopside and augite (n = 104) and orthopyroxene classify as clinoenstatite and pigeonite (n = 303). (**d**) Euhedral plagioclase crystal with visible zoning and twinning under crossed-polarised light (XPL). (**e**) Subhedral clinopyroxene crystal overgrown by titanomagnetite (opaque phase) under XPL. (**f**) Exsolution lamellae of titanomagnetite in a subhedral clinopyroxene crystal (BSE image). (**g**) Clinopyroxene and titanomagnetite glomerocryst (BSE image).

Lavas from the 1963 eruption of Agung are basaltic andesite to andesite in composition (Fig. 2). They are dark grey, hypocrystalline, porphyritic, and moderately vesicular. Samples are characterised by up to ~40 vol.% phenocrysts, whose assemblage is dominated by plagioclase, orthopyroxene, titanomagnetite, and clinopyroxene set in a glassy, microlitic groundmass (Fig. 3).

Plagioclase phenocrysts are euhedral to subhedral, ≤2 mm in size, with a compositional range of An_42–96_, (average An_66 ± 10_; 2σ, n = 401; Fig. 3a,b). They are usually twinned and display complex zonation patterns as well as evidence for dissolution and overgrowth (Fig. 3d). Orthopyroxene is euhedral to subhedral, ≤1.5 mm in size, commonly zoned, and has compositions that range from Wo_0_En_47_Fs_25_ to Wo_16_En_72_Fs_42_. Orthopyroxene classifies as clinoenstatite and has Mg numbers (Mg#) ranging from 53 to 74 (average = 69 ± 2; 2σ, n = 303; Fig. 3c). Clinopyroxene is anhedral, less abundant than orthopyroxene, ≤1 mm in size and mostly classifies as augite in the range Wo_37_En_40_Fs_13_ to Wo_45_En_46_Fs_20_ (Fig. 3c). Phenocrysts of clinopyroxene have Mg# between 67 and 77 (average = 72 ± 2; 2σ, n = 104). Both orthopyroxene and clinopyroxene display overgrowth and exsolution of titanomagnetite (Fig. 3d–g). Titanomagnetite is also present as occasional phenocrysts, usually ≤1 mm, and commonly in the form of crystal clots where it occurs together with other, more abundant crystal phases. The groundmass consists of plagioclase, clino/orthopyroxene, and titanomagnetite microlites and glass.

#### Batur

**Figure 4:**
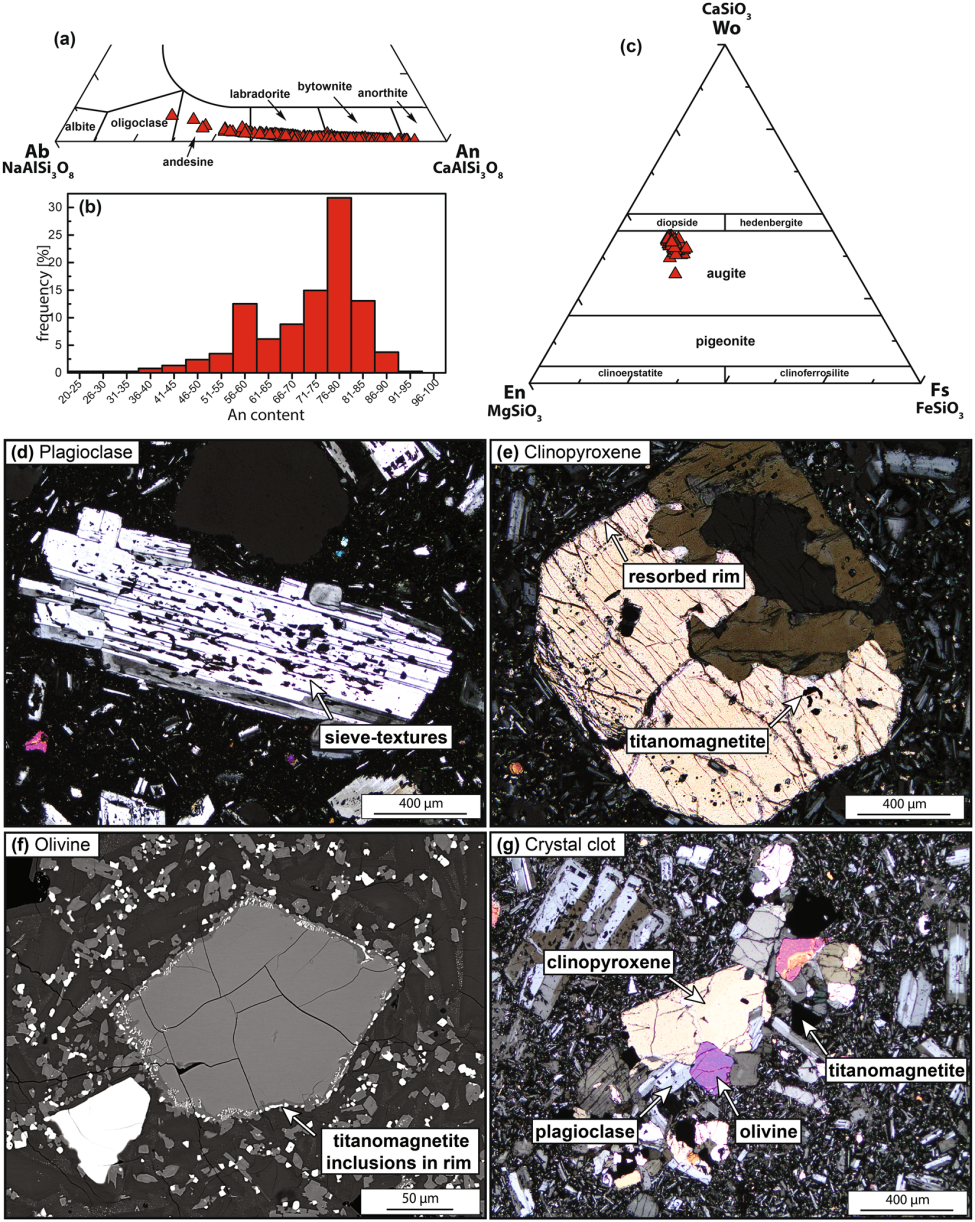
Mineral chemistry and petrography for Batur. (**a**) Compositions of plagioclase from the 1963 and 1974 Batur eruptions (n = 375), which classifies as oligoclase to anorthite. (**b**) Frequency of anorthite content in Batur plagioclase in the range of An_20–92_, with a peak at An_76–80_. (**c**) Compositions of clinopyroxene from the 1963 and 1974 eruptions of Batur. Clinopyroxene classifies as augite (n = 86). (**d**) Euhedral plagioclase crystal with twinning and internal sieve textures under crossed-polarised light (XPL). (**e**) Euhedral clinopyroxene crystal with titanomagnetite overgrowth that likely followed after a dissolution event (XPL). (**f**) Olivine crystal showing a corona of mainly titanomagnetite (BSE image). (**g**) Crystal clot of clinopyroxene, olivine, plagioclase, and titanomagnetite (XPL).

Lavas from the 1963 and 1974 eruption of Batur are dark grey, porphyritic, and moderately vesicular basaltic andesites (Figs 2 and 4). All samples contain plagioclase, clinopyroxene, olivine, and titanomagnetite as the main mineral phases, which together total up to ~40 vol.% phenocrysts set in a glassy, microlite-bearing groundmass. The most abundant mineral phase is plagioclase, which varies in composition from An_26_ to An_92_ with an average of An_71±11_ (2σ, n = 375; Fig. 4a,b). Plagioclase is normally euhedral to subhedral, ≤3 mm in size, and commonly displays sieve textures, frequent twinning and patchy zoning in back scattered electron (BSE) images (Fig. 4d). Clinopyroxene crystals show Mg# that range from 66 to 76 (average = 73 ± 2; 2σ, n = 86) and a compositional range from Wo_32_En_40_Fs_14_ to Wo_43_En_47_Fs_21_, classifying them as augite (Fig. 4c). Phenocrysts of clinopyroxene are smaller in size than plagioclase (≤2 mm), generally zoned, and show sieve textures and overgrowth by titanomagnetite and olivine (Fig. 4e). Olivine phenocrysts are less abundant than plagioclase and clinopyroxene, ≤1.5 mm in size, and show reaction rims that are rich in titanomagnetite microcrysts (Fig. 4f). The compositional range of olivine is Fo_45–71_ with an average of Fo_68 ± 3_ (2σ, n = 136). The groundmass consists of plagioclase, clinopyroxene, olivine and titanomagnetite microlites, which occasionally form microlite crystal clots (Fig. 4g).

### Thermobarometry

To determine the depth of crystallisation of pyroxene and plagioclase, we used the thermobarometry models that are summarised in Geiger *et al*.^21^ (for details see below). Equilibrium conditions of mineral-melt pairs are a prerequisite for mineral-melt (thermo-)barometric models and full details of the equilibrium tests conducted in this study can be found in the Supplementary Information. Several thermobarometric models were applied to equilibrium mineral-melt pairs, as summarised in Table 1 and in the Methods. A summary of the results obtained using the various models  is presented below and can be found in full detail in the Supplementary Information.

**Table 1:** Thermobarometric models used in this study and their associated uncertainties.

Abbreviation	Reference	Standard error of estimate
PT08Al	Putirka^54^ Eq. 32c	±150 MPa
PT08Jd	Putirka^54^ Eq. 30 & 33	±33 °C and ±170 MPa
PT08Nim	Putirka^54^ Eq. 32b	±260 MPa
PTPlag	Putirka^54,60^	±36 °C and ±247 MPa
KB08	Kelley and Barton^62^	±110 MPa (1σ)

#### Clinopyroxene (-melt) thermobarometry

For the 1963 eruption of Agung, applying the PT08Al formulation (see Table 1) to equilibrium clinopyroxene and matrix glass pairs yields crystallisation pressures of 364 to 905 MPa. These pressures translate to crystallisation depths between 11 and 27 km. Employing the PT08Jd model on the same set of equilibrium clinopyroxene-melt pairs yields a pressure range from 389 to 1128 MPa, which translates to crystallisation depths between 12 and 33 km. Pressures of 341 to 820 MPa are derived for clinopyroxene using the PT08Nim model and these values correspond to crystallisation depths between 10 and 24 km. This is a more limited pressure-depth range, but largely overlaps with the results from PT08Al and PT08Jd.

When using PT08Al on clinopyroxene-matrix pairs from the 1963 Batur eruption, the resulting pressures range from 262 to 663 MPa, which translates to between 9 and 23 km depth. In turn, PT08Jd results in a pressure range for the equilibrium crystal-matrix pairs of 331 to 583 MPa, which corresponds to a crystallisation depth of 11 to 20 km. Employing the PT08Al formulation on clinopyroxene and equilibrium whole-rock data from de Hoog *et al*.^12^ resulted in a pressure range from 151 to 542 MPa. This translates to a crystallisation depth of between 5 and 19 km. The PT08Jd model, in turn, calculates pressures for these crystal-whole rock pairings of between 320 and 576 MPa, or 11 and 20 km depth.

Applying the PT08Al formulation on the clinopyroxene from the 1974 Batur lavas in combination with suitable melt compositions gives pressures that range from 302 to 637 MPa (10 to 22 km depth) for the whole-rock composition from this study and from 284 to 620 MPa (10 to 21 km depth) for the whole-rock composition from de Hoog *et al*.^12^. The results from PT08Jd yield similar pressures of 352 to 670 MPa (12 to 23 km depth) for whole-rock from this study and pressures from 357 to 675 MPa (12 to 23 km depth) for whole-rock data from de Hoog *et al*.^12^. In addition, PT08Nim was used to verify the results from clinopyroxene-melt thermobarometry for Batur. For 1963 clinopyroxene, PT08Nim calculates pressures between 277 and 498 MPa (11 to 20 km depth), whereas for clinopyroxene from the 1974 eruption, pressures between 239 and 615 MPa are obtained (8 to 21 km depth). Results from PT08Nim are thus in broad agreement with depth obtained from clinopyroxene-melt barometers.

#### Plagioclase-melt thermobarometry

The PTPlag formulation in combination with rasterized matrix compositions yield crystallisation pressures between 131 and 200 MPa for the 1963 Agung eruption, which correspond to a depth of ~4.5 to 7 km. Resulting pressures for equilibrium mineral-melt pairs from the 1963 Batur eruption range from 79 to 147 MPa, corresponding to a crystallisation depth of ~3 to 5 km. Plagioclase-melt thermobarometry on equilibrium mineral-melt couples from the 1974 Batur eruption resulted in pressures between 104 and 235 MPa, which translates to a depth of ~4 to 8 km. We note that rasterized matrix compositions are crucial in order to obtain reliable equilibrium results for the PTPlag formulation (see Methods).

#### Olivine-plagioclase-augite-melt (OPAM) boundary barometry

Applying the KB08 model to the equilibrium melts used for clinopyroxene-melt and plagioclase-melt thermobarometry for Agung and Batur resulted in a wide range of pressures from 57 to 677 MPa. This corresponds to a depth range of 2 to 23 km. These values are in broad agreement with depth estimates obtained from the other thermobarometric models used in this study.

### Helium isotopes

The helium isotope ratio (^3^He/^4^He, air-corrected) of a pyroxene separate from the 1963 Agung lava was determined at 8.62 ± 0.39 R_A_ (see Supplementary Table S1), which is consistent with previous ^3^He/^4^He values recorded in the region^22^ and is characteristic for mantle-like signatures of 8 ± 1 R_A_.

### Oxygen isotopes

Oxygen isotope ratios are reported in standard delta notation for a total of 15 crystal separates of olivine, clinopyroxene and plagioclase from the studied eruptions (Supplementary Table S2). For the 1963 eruption of Agung, clinopyroxene and plagioclase yielded average δ^18^O values of 5.5‰ (n = 3, stdev = 0.24) and 6.2‰ (n = 2, stdev = 0.31), respectively. Olivine from the 1963 eruption of Batur show an average δ^18^O value of 4.8‰ (n = 2, stdev = 0.06), whereas clinopyroxene and plagioclase record δ^18^O values of 5.2‰ (n = 1) and ~5.8‰ (n = 2, stdev = 0.17), respectively. For the 1974 eruption of Batur, clinopyroxene and plagioclase yielded average δ^18^O values of 5.3‰ (n = 2, stdev = 0.41) and 5.7‰ (n = 2, stdev = 0.37), respectively. Notably, plagioclase average values are higher in all cases than the clinopyroxene and olivine separates from the same samples.

## Discussion

### Magma storage below Agung

The combined results from clinopyroxene-melt, single clinopyroxene, plagioclase-melt, and OPAM thermobarometry suggest a poly-baric magma storage system beneath Agung volcano. Clinopyroxene-melt and single clinopyroxene models are in good agreement with each other and record a major level of crystallisation at ~18 to 22 km depth, which is around the MOHO in the region. Helium and oxygen isotope data for pyroxene from Agung point towards a dominantly mantle origin of the pyroxene crystals (see below), and do not record a significant crustal input.

Plagioclase phenocrysts record another dominant level of crystallisation between 3 and 7 km depth, which appears to be located around the boundary between the upper sedimentary crust and the underlying oceanic-type basement^16^. Plagioclase separates also record elevated oxygen isotope values relative to clinopyroxene and olivine separates, in line with mild crustal additions to the host magma from which these crystals grew. This upper crustal crystallisation level is notably consistent with Interferometric Synthetic Aperture Radar (InSAR) measurements, which detected a magma reservoir between 2 and 4 km depth beneath Agung^23^. The depth attained from InSAR uses the surrounding plain as a reference point, whereas barometry takes the total overload, including the volcanic edifice (~3 km), into account. Depth estimates attained from barometry for the shallow magma storage level therefore overlap with the InSAR results when correcting for the different reference levels.

Further characterisation of the magma system is possible by combining the results from thermobarometry with petrographic and geochemical data. Lavas from the 1963 eruption of Agung changed in composition throughout the eruption from andesite to basaltic-andesite^9^. In addition to an increase in MgO and a decrease in SiO_2_, an increase in compatible trace elements was observed^2^, suggesting that the 1963 Agung eruption was associated with magma recharge at depth^2,9^. This recharge event would have triggered magma mixing, consistent with frequently observed sieve-textures in albite plagioclase that indicate interaction with a hotter or more Ca-rich melt (cf.^24^). A basaltic melt was thus likely injected into the upper-crustal 1963 Agung holding system at 3 to 7 km depth prior to eruption and triggered the 1963 event (cf.^25^).

### Magma storage below Batur

The thermobarometric results from clinopyroxene, plagioclase, and whole rock in the eruptive products of the 1963 and 1974 eruptions of Batur also indicate a polybaric magma supply system. Thermobarometry of clinopyroxene from the 1963 eruption reveals a dominant storage level between 12 and 18 km depth, whereas plagioclase-melt thermobarometry points to additional shallower storage between 2 and 4 km depth. Results for mineral phases from the 1974 eruption show similar, albeit slightly deeper crystallisation depths of 15 to 19 km for clinopyroxene and 3 to 5 km for plagioclase. For both eruptions, the results from the applied thermobarometric models are in good agreement, underscoring the validity of the results obtained. Similar to Agung, a shallow magma reservoir has also been detected at Batur through geophysical methods, in particular by seismic activity, which shows increased earthquake clusters at depths of between 1.5 and 5 km below the volcano’s summit^8^. Additionally, oxygen isotope data for olivine point to crystallisation from mantle-like melts, whereas clinopyroxene and plagioclase show slightly evolved values.

The magma storage levels below Batur are at similar depths to those beneath Agung. However, in contrast to Agung, Batur lavas contain olivine and lack orthopyroxene. Moreover, Batur also exhibits shorter repose times, and generates less violent eruptive events than Agung. Additionally, recent InSAR data reveal precursory inflation and deflation at Agung while Batur remained stable during the measurement timeframe^26^. The balance of evidence therefore suggests that although the magmatic plumbing systems beneath the two volcanoes are analogous in their broad architecture, they are in fact separate entities. We note, however, that at the time of writing, Agung was in a state of unrest and seismicity appears to have originated deep below Batur volcano before moving laterally and upward towards Agung’s plumbing system. Although our data do not support a connection between the two plumbing systems in 1963 and 1974, the recent seismic data from late 2017 point to a possible temporary connection after all^27^.

In this context, it should be noted that the recent Batur lavas may not represent the full magmatic system underlying the Batur Volcanic Field as they do not span the whole compositional range of older eruptive suites, i.e. felsic compositions are restricted to earlier caldera-forming events. In fact, some of Batur’s dacitic lavas were found to have originated from near closed-system fractionation of evolved shallow magma pockets^13^, which suggests that isolated but evolved magma bodies may exist beneath the Batur Volcanic Field. If present and if intersected by ascending mafic magma, such felsic magma pockets could have considerable impact on the style of eruptions^cf.^^3,13,15,20,21,28^.

### Oxygen isotope temperature determinations and equilibrium assessment

To further assess the mineral oxygen isotope data, we first employ mineral-mineral equilibria between clinopyroxene and plagioclase mineral pairs (i.e. ∆_clinopyroxene-plagioclase_). Minerals in isotopic equilibrium from a given rock suite are related to each other by constant ∆ and hence constant temperature^29^. This is the case for the averaged mineral-melt pairs in this study from the three investigated eruptions, which correlate positively with respect to their δ^18^O_pyroxene_ versus δ^18^O_plagioclase_ (R^2^ = 0.8). Indeed, the ∆_clinopyroxene-plagioclase_ from the Agung 1963, Batur 1963, and Batur 1974 lavas are relatively small at 0.7, 0.6, and 0.4‰, respectively. Using the equations of Chiba *et al*.^30^ and adopting an anorthite content of 70 mol.%, we arrive at equilibrium crystallisation temperatures in the range 1000 to 1100 °C for Agung 1963 and Batur 1963, which is in excellent agreement with the clinopyroxene-melt thermometry results (see Supplementary Information). The Batur 1974 mineral pairs have a smaller ∆_clinopyroxene-plagioclase_ of 0.4, which when combined with an anorthite content of 85 mol.%, indicates equilibrium crystallisation at ca. 1150 °C, close to the temperature calculated using clinopyroxene-melt thermometry (see Supplementary Information).

Although we have established that Bali pyroxene and plagioclase crystallised under equilibrium conditions, the results of multiple analysis of a given mineral type from the same sample sometimes reveal differences (Supplementary Table S2). This difference (max 0.6‰) is larger than the analytical precision (see Methods) and could point to minor heterogeneity among clinopyroxene grains, variable alteration along mineral fractures or the presence of low δ^18^O inclusions, such as magnetite^31^. Indeed, a large degree of oxygen isotopic heterogeneity among the clinopyroxene population of Merapi volcano, Central Java, was recently identified based on intra-crystal spot analysis with variations exceeding 1‰ (ref.^32^), implying that small-scale magmatic heterogeneities may also exist at Batur and Agung.

To estimate the magma δ^18^O values from the mineral data we assume δ^18^O fractionation of −0.2‰ for plagioclase, +0.3‰ for pyroxene^31^, and +0.4‰ for olivine^33^. On the basis of these fractionation factors, Batur olivine with δ^18^O values of 4.8 and 4.9‰ reflect primitive magma compositions of ca. 5.2‰. These δ^18^O values overlap within error with the lower end of the δ^18^O range obtained for olivine from upper-mantle peridotite xenoliths^34^ (5.0 to 5.4‰;) and the established MORB range^35^ (5.7 ± 0.3‰). We note that Batur olivine contain a minor amount of low δ^18^O inclusions (magnetite), which makes the calculated melt values minimum estimates only (cf.^32^). Clinopyroxene and plagioclase from Agung and Batur, in turn, crystallised from magma with δ^18^O values of 5.3 to 6.1‰ (average = 5.7‰) and 5.3 to 6.2‰ (average = 5.7‰), respectively. The magma δ^18^O values for Agung and Batur are thus within error of each other. These data, moreover, overlap with the primary mafic magma δ^18^O value recently reported for Merapi volcano in Central Java^32^ (average = 6.1‰ ± 0.4 (2σ)). Our new data therefore point to a mantle-dominated system beneath Batur whereby olivine crystallised from mafic melts at depth. Clinopyroxene and plagioclase at both Agung and Batur, in turn, crystallised from slightly more evolved magma during storage in crustal magma reservoirs. This model is consistent with the thermobarometric data presented here as well as arc-wide InSAR data that confirm upper crustal magma storage (see below). Notably, the δ^18^O values of the Agung and Batur basaltic-andesites do not show evidence for extensive slab enrichment of the mantle wedge or deep crustal assimilation, but support a small degree of upper crustal magma-crust interaction (see also below).

### A model for Agung and Batur

**Figure 5:**
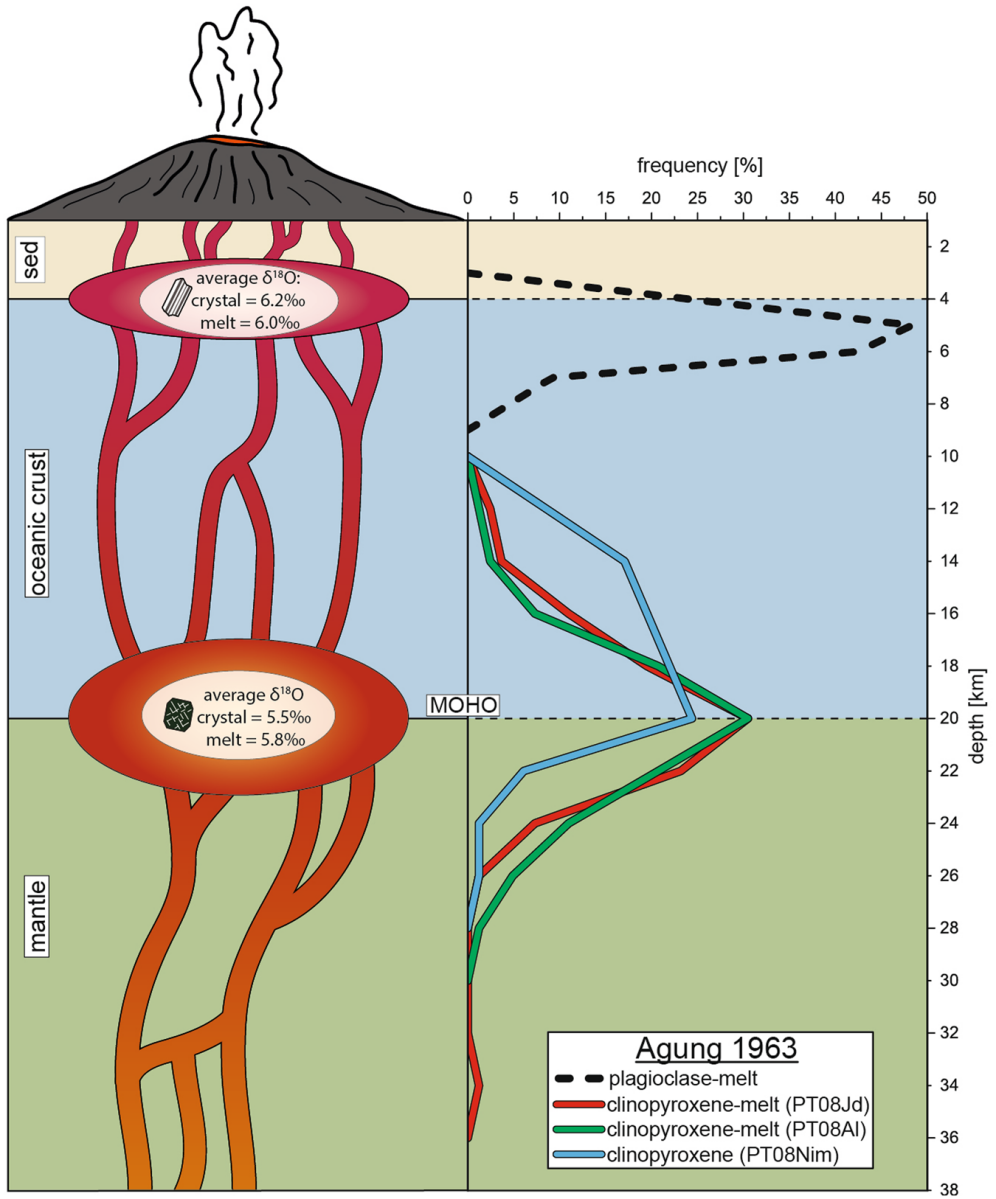
Magma plumbing beneath Agung. A possible model for the plumbing system beneath Agung based on mineral-melt thermobarometry of the 1963 lavas. Two major magma storage regions are apparent in the frequency plot: one at 18 to 22 km depth, around the MOHO, and another at 3 to 7 km depth, likely at the boundary between the upper crustal sedimentary units and the tectonised oceanic-type middle to lower crust. The calculated melt δ^18^O values based on clinopyroxene and plagioclase mineral analysis average at 5.8‰ for the lower reservoir and 6.0‰ for the shallow storage level.

**Figure 6:**
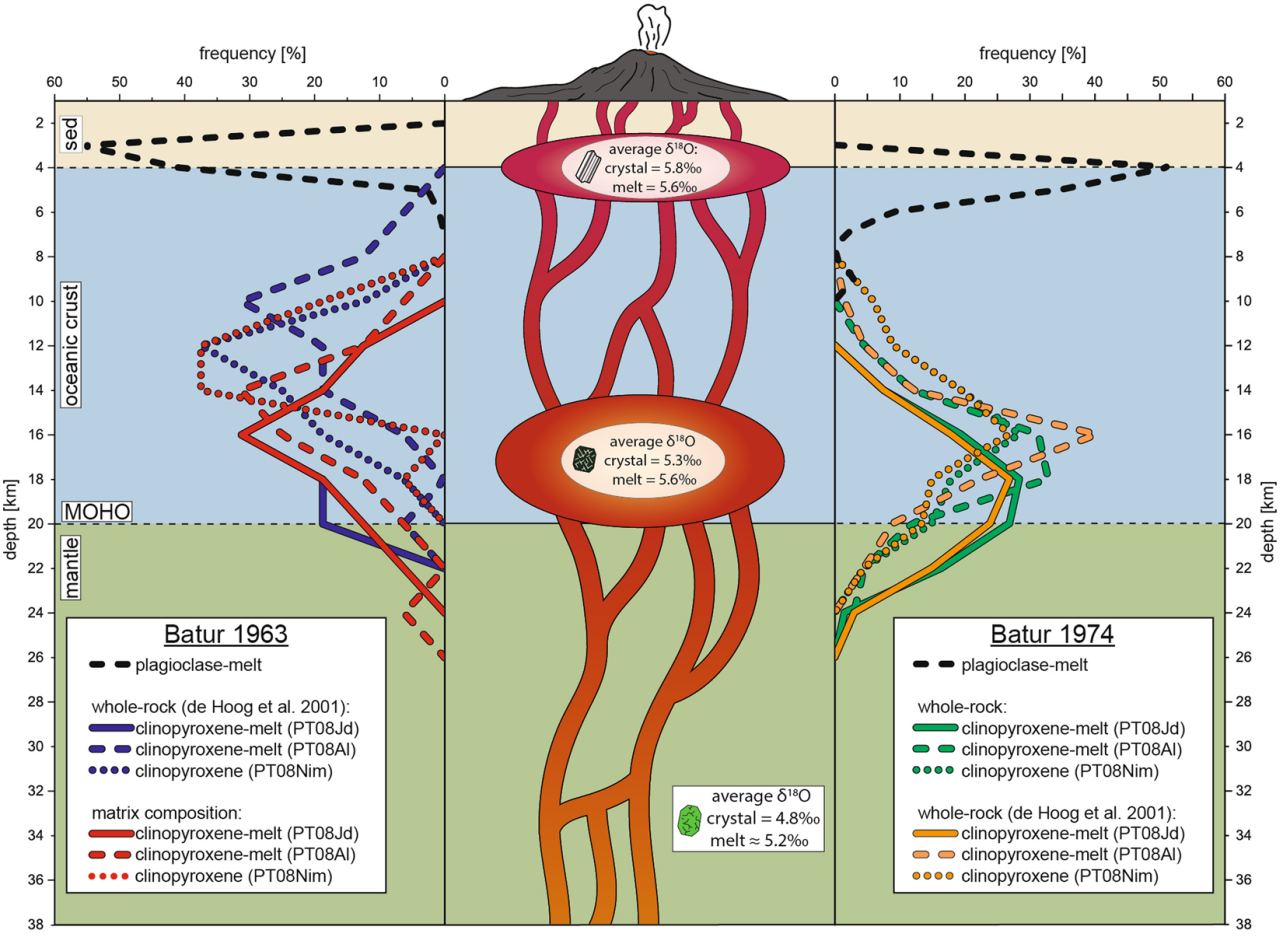
Magma plumbing beneath Batur. A possible model for the plumbing system beneath Batur based on thermobarometry frequency plots. Two main magma storage regions are recorded by each of the eruptions. For the 1963 eruption, one storage level resided at 12 to 18 km depth and another at 2 to 4 km depth. For the 1974 eruption, the deeper reservoir is located at 15 to 19 km depths whereas a shallow storage region is found at 3 to 5 km depth. Batur’s plumbing system is reflected in the calculated melt δ^18^O values based on olivine, clinopyroxene, and plagioclase mineral analysis. Olivine indicates mantle-dominated deep input to the system with a primitive melt δ^18^O value of ≥5.2‰. Clinopyroxene and plagioclase, in turn, crystallised in crustal reservoirs from a slightly more evolved melt with δ^18^O values of ca. 5.6‰.

Application of a set of suitable thermobarometric models to clinopyroxene from the 1963 eruption of Agung shows a major crystallisation and hence magma storage level between 18 and 22 km depth, which is around the MOHO level in the region (Fig. 5). Crystallisation depths of 12 to 18 km for the 1963 and 15 to 19 km for the 1974 eruption of Batur suggest a major reservoir around and maybe slightly above the MOHO beneath Batur (Fig. 6). Shallow-level reservoirs, in turn, are recorded by plagioclase, with depths of 3 to 7 km for the 1963 eruption of Agung and of between 2 to 4 km for the 1963 Batur eruption and between 3 to 5 km for the 1974 Batur eruptive event (Figs 5 and 6). Thus, for both volcanoes, the results from plagioclase-melt thermobarometry point to a magma reservoir at ~4 km depth, in line with recent InSAR results and seismic data^8,26^. This shallower level notably coincides with the transition between the sedimentary rocks that comprise the uppermost crust in Bali and the underlying tectonised oceanic basement. The density contrast within the crust at this level may be a cause for magma to stall. We therefore argue that the mantle-crust boundary and intra-crustal lithology changes play a major role in controlling the level of magma storage beneath Agung and Batur (cf.^36^). However, even though magma storage is found at similar depths for Agung and Batur, mineralogical and geophysical evidence points towards two separate magma supply systems for the 1963 event (see above).

### Magma storage along the Java-Bali segment of the Sunda arc

**Figure 7:**
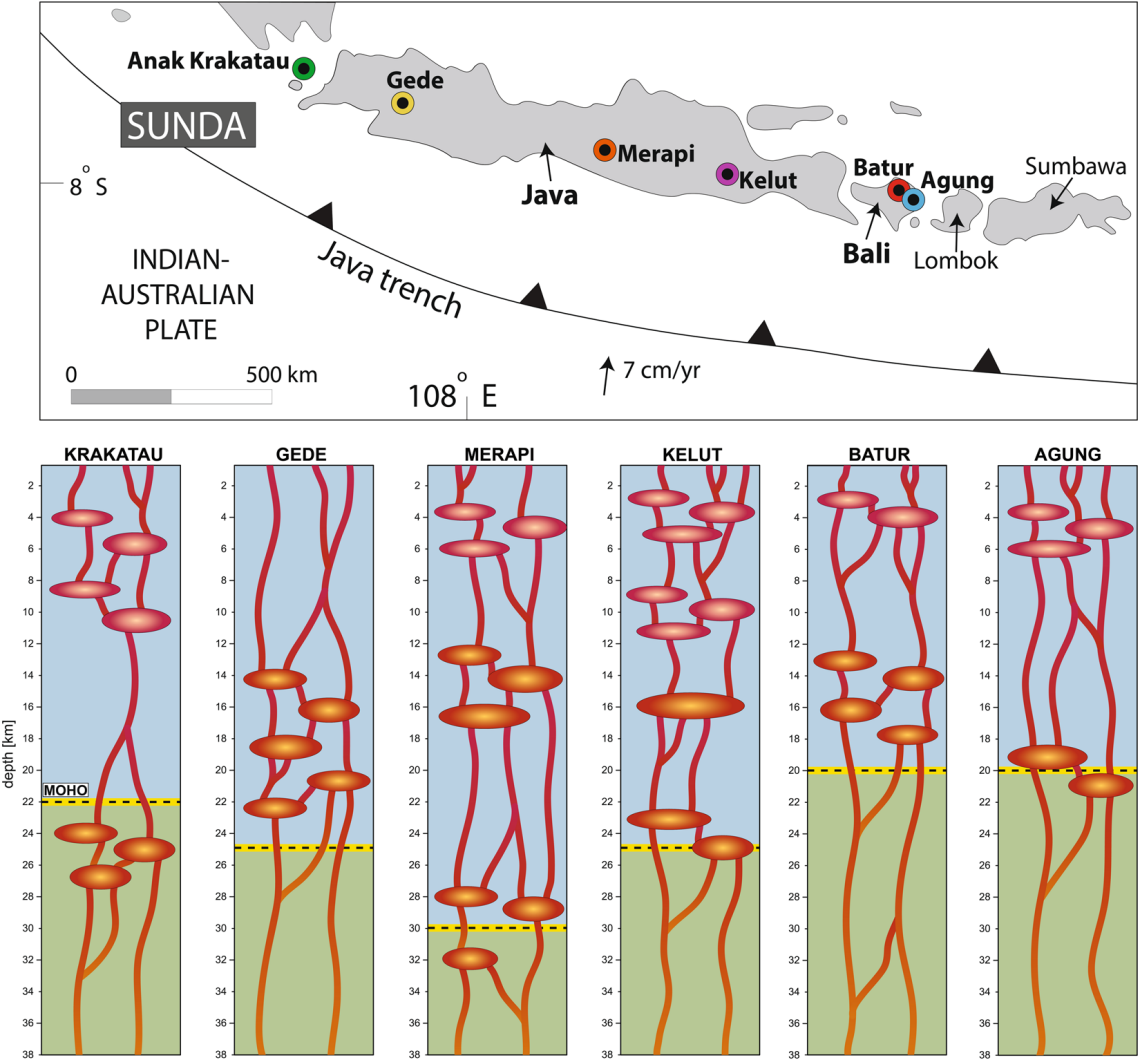
Magma storage along the Java-Bali segment of the Sunda arc. Regional comparison between the main crystallisation levels of plagioclase, pyroxene and amphibole as revealed by thermobarometry for Krakatau (plagioclase and clinopyroxene), Gede (two-pyroxene), Merapi (plagioclase, amphibole, clino- and orthopyroxene), Kelut (plagioclase, clinopyroxene and amphibole), Batur and Agung (plagioclase and clinopyroxene; this study). See Supplementary Table S3 for references. A general model for crystallization within Sunda arc volcanoes involves clinopyroxene, amphibole and high-An plagioclase crystallisation in lower crustal magma reservoirs (ca. 110 to 760 MPa; ≥20 km depth). In a number of cases crystallization of pyroxene appears to also occur in the mid-crust (ca. 50 to 380 MPa, equivalent to ~15 to 10 km), and thus mainly below the fertile sedimentary cover that forms the top ≤10 km of the Java crust^32,36,43–45,51^. Minor pyroxene crystallization may finally take place in the top few kilometres of the upper crust in some systems, such as Merapi. In turn, the main crystallization level for medium An plagioclase appears to be constrained to the top 10 km of the crust in most cases, i.e., that is within the shallow sedimentary portion of the crust^36,43–45,51^ (ca. 20 to 190 MPa). These shallow magma reservoirs may give only limited warning time prior to an eruption and will act as sites for intense magma degassing due to low volatile solubilities at such shallow pressures ^e.g.^^50^.

Mineral barometry on plagioclase, pyroxene, and amphibole has recently become available from an increasing number of volcanic centers along the Java-Bali segment of the Sunda arc, including Anak-Krakatau volcano, Gede volcano, Merapi volcano, and Kelut volcano (see Supplementary Table S3 for references). The crystallization depths inferred by petrological means can be compared to the results of geophysical investigations carried out in Java such as seismic, magnetotelluric and long-offset transient electromagnetic experiments (e.g. at Anak-Krakatau, Merapi and Lawu volcanoes). These studies imply aseismic zones situated at 1.5 to 2.5 km depth at these volcanoes, indicating the existence of shallow magma reservoirs with likely regular magma supply from deeper levels^37–41^. Most recently, ground displacements via InSAR measurements on six volcanoes along the Sunda Arc (Sinabung and Kerinci in Sumatra, and Slamet, Lawu, Lomongan on Java and Agung on Bali) detected shallow magma reservoirs at ~1 to 3 km depth^23,26,42^, which the authors relate to extensional and strike-slip settings caused by the intra-arc stress regime. Shallow magma storage is hence increasingly detected with both petrological and independent geophysical methods along the Sunda arc. The available studies thus point to complex supply systems feeding these volcanoes, involving multi-stage magma storage in the crust prior to eruption at the surface (Fig. 7). This realization is consistent with other geological and petrochemical information on Sunda arc storage systems, such as crustal structure, crystal size distribution (CSD), as well as radiogenic isotopes on plutonic inclusions that point to frequent poly-baric crustal magma storage at these volcanoes (e.g.^43–45^).

The concept of poly-baric storage beneath the Sunda arc volcanoes is, moreover, consistent with recent propositions of shallow crustal cold zones as a major source of intermediate to felsic magmas in arcs^46^ in addition to deeper crustal hot zones (e.g.^47^). Not only does it seem that such upper crustal cold zones are more widespread in arc settings than previously thought, but likely they are also the sites were the final conditioning of magma prior to eruption is accomplished (e.g.^48^). Specifically, the consistency of results from diverse methods in favour of shallow-level storage in the Sunda arc provides a plausible link between magma ascent and the relatively widespread evidence for late-stage (shallow-level) crustal differentiation and assimilation in many Sunda arc volcanoes^44,45,49–51^. Shallow magma storage may thus facilitate favourable conditions for magma differentiation, which in turn, could drive magma evolution to more felsic compositions. Furthermore, these shallow magma reservoirs may act as sites for intense magma degassing for residing and replenishing magmas due to low volatile solubilities at such shallow crustal levels. The shallow depth of these reservoirs may provide limited advanced warning before eruption, as was recently the case for Kelut volcano in East Java in February 2014. After only a few days of unrest, Kelut erupted violently over the course of a few hours from an upper crustal magma reservoir^50^. The intensity of the 2014 Kelut eruption, along with the exclusively shallow crustal and short-lived seismic warning signals, underlines the hazardous nature of shallow magma reservoirs in arc-type plumbing systems.

## Methods

### Whole Rock and Mineral Chemistry

This study is based on whole rock lava samples from the 1963 eruption of Agung (n = 6) and the 1963 & 1974 eruptions of Batur (n = 2 & 3, respectively). Representative samples were analysed for their bulk rock major and trace element composition (see Supplementary Information for details). Mineral and matrix compositions as well as backscattered electron (BSE) images of selected minerals were acquired using the Jeol JXA8530F Hyperprobe Field Emission Gun Electron Probe Micronanalyser (FEG-EPMA) at Uppsala University (UU), Sweden, which is equipped with five spectrometers. Measurements were conducted under standard operating conditions of 15 kV accelerating voltage and 10 nA beam current with counting times of 10 s on peaks and 5 s on ± background. Beam diameter was set to 1–5 µm for mineral analysis and 10 µm for matrix analyses (including microphenocrysts). Iron content is reported as FeOt. The five spectrometers were calibrated using wollastonite for Ca and Si, pyrophanite for Mn and Ti, magnesium oxide for Mg, orthoclase for K, albite for Na, aluminium oxide for Al, fayalite for Fe and chromium oxide for Cr. In order to maintain data quality, analytical precision was verified using Smithsonian Institute mineral standards. The resulting data set for Agung volcano consists of 401 point analyses of plagioclase from 125 individual crystals, 303 single point analyses from 145 orthopyroxene crystals, 104 point analyses of clinopyroxene from 49 crystals as well as six 5 × 5 step grid analyses of the matrix (1 step = 10 µm). For Batur, the data set comprises 375 point analyses from 121 individual plagioclase crystals, 86 point analyses from 81 clinopyroxene crystals, 136 single point analyses from 39 olivine crystals as well as seven 5 × 5 step grid analyses of matrix (see Supplementary Information for full mineral and groundmass composition data set).

### Clinopyroxene(-melt) thermobarometry

We applied several thermobarometric models to the derived Agung and Batur mineral data and lava compositions. For clinopyroxene-melt equilibrium thermobarometry, the two models with the highest precision and the least systematic error are the formulations by Putirka *et al*.^52,53^, calibrated for anhydrous and hydrous systems, respectively. Both models are based on the jadeite-diopside/hedenbergite exchange between clinopyroxene and the associated melt (Eq. (A) and (B) in Putirka *et al*.^53^) and have a standard error of estimate (SEE) of ±33 °C for predicted temperature and ±170 MPa for pressure. We used a recent re-calibration of these models by Putirka^54^ (Eq. 30 & 33) that incorporates H_2_O as input parameter in order for the model to be yet better applicable to hydrous systems. This most recent version of the model will be referred to as PT08Jd.

Another approach was introduced by Putirka^54^ and is based on Al partitioning between clinopyroxene and melt (Eq. 32c in Putirka^54^). This model requires H_2_O as well as temperature input and the latter can be provided by the Putirka *et al*.^53^ model, thus representing a superior approach to other thermobarometric formulations (e.g. PT08Jd) for hydrous systems and has an SEE of ±150 MPa. However, this model is not yet routinely used and has therefore not undergone as much testing as the Putirka *et al*.^53^ model. This model will be referred to as PT08Al in the present study.

A widely used barometer based exclusively on clinopyroxene composition is the model by Nimis^55^ that was later extended by Nimis & Taylor^56^. This approach does not require an associated melt and can predict pressures from clinopyroxene compositions alone when an input for temperature is available. However, when applied to hydrous systems, this model tends to systematically underestimate pressure^54^. A re-calibration by Putirka^54^ (Eq. 32b) requires H_2_O content as an input parameter, making it applicable to hydrous systems and removing the systematic error, but rendering the approach slightly less accurate than other methods, with an SEE of ±260 MPa (ref.^54^) (versus ±200 MPa for the original approach). This model will be referred to as PT08Nim.

Equilibrium conditions of mineral-melt pairs are a prerequisite for all mentioned (thermo-) barometric models. A possible method to assess equilibrium of clinopyroxene-melt couples is the K_D_(Fe-Mg) exchange coefficient between clinopyroxene and the nominal melt. This method is in regular use (e.g.^21,28,36,57^) and assumes equilibrium conditions if a clinopyroxene-liquid pair falls into the K_D_(Fe-Mg) = 0.28 ± 0.08 envelope^54^. However, this approach does not take into account other important clinopyroxene exchange equilibria, namely Na-Al or Ca-Na exchange. It is therefore recommended to perform a second equilibrium test by comparing predicted versus observed mineral components^58–60^ (e.g. DiHd, EnFs, CaTs, Jd;). A close match (within ± 0.10) of the predicted versus observed mineral components is needed to validate the findings obtained from the K_D_(Fe-Mg) test.

### Plagioclase-melt thermobarometry

The most recent plagioclase-melt thermobarometry approach by Putirka^54^ (Eq. 25a) is an improved version of the model described by Putirka^60^. The model is calibrated for hydrous systems, and thus requires H_2_O input. It predicts pressure with an SEE of ±247 MPa and temperature with an SEE of ±36 °C (ref.^54^). Potential mineral-melt pairs for plagioclase-liquid thermobarometry can be chosen by applying the K_D(An-Ab)_ equilibrium test as described by Putirka^54^. This test is based on Ab-An exchange and the K_D_ has been shown to be largely independent with respect to pressure, temperature and H_2_O variations. Only data points falling into the range K_D(An-Ab)_ = 0.10 ± 0.05 for T < 1050 °C or 0.27 ± 0.11 for T ≥ 1050 °C are considered to be in equilibrium with the chosen associated melt and are used for further calculations.

An additional test for the validity of the selected mineral-melt pairs is a comparison of predicted temperatures with plagioclase saturation surface temperatures of the nominal melt (Eq. 26 in Putirka^54^). If the saturation surface temperature, which is the lowest possible temperature of the melt before the onset of plagioclase crystallisation, closely matches with the calculated temperature from mineral-melt compositions (Eq. 24a in Putirka^54^), equilibrium of the mineral-melt pair is indicated.

We employed rasterized matrix analysis for determination of the nominal melt in the plagioclase thermobarometry model. This is because whole rock compositions are frequently employed as nominal melts, but can produce imprecise results due to the fact that whole rock chemistry is an averaged magma composition. It is thus not always representative of the melt from which late-stage plagioclase crystallised (e.g.^54^). Rasterized matrix compositions, on the other hand, appear to be a better fit for the final melt in equilibrium with plagioclase in our study since they produce the more reliable results.

### Olivine-plagioclase-augite-melt (OPAM) boundary barometry

Yet another method to assess crystallisation temperatures and pressures employs phase relations for the olivine-plagioclase-clinopyroxene cotectic boundary (OPAM) on whole rock or glass compositions. This approach was first developed by Yang *et al*.^61^ and later modified by Kelley & Barton^62^ and is independent of mineral compositions. It can therefore be used as an independent test with an accuracy of ±110 MPA (1σ) and will be referred to as KB08.

### Input parameters

Some of the mentioned thermobarometric models require water contents besides the input of mineral and melt compositions. Arc magmas generally have pre-eruptive volatile (i.e. H_2_O, CO_2_ and S) contents of ~2.2 wt% to ~5.4 wt%, with an average of ~3.8 wt% (ref.^63^). Volatile contents in melt inclusions in lavas from Agung have a reported average volatile content of 4.3 wt%, most of which is water^9^. For Batur magmas, estimates of pre-eruptive H_2_O content range from 3 to 6 wt% (ref.^13,15^). Hence an average of ~4 wt% H_2_O is used here, which appears to be a reasonable approximation for both volcanoes (cf.^64^).

The equilibrium test for clinopyroxene-melt thermobarometry is based on Fe/Mg partitioning, and therefore requires an estimate of the Fe^3+^/Fe_total_ ratio in the melt. Global arc magmas have Fe^3+^/Fe_total_ ratios of 0.18 to 0.32, as measured in basaltic melt inclusions^63,65^. A mid-range value of 0.25 is used here, which we consider a suitable estimate for our calculations.

In order to be able to convert the obtained pressure values to depth, rock densities of the volcanoes’ underlying stratigraphy are required. Seismic studies have revealed that the stratigraphy south of Bali in the Lombok forearc basin is characterised by a succession of sediments (<4 km), underlain by oceanic crust to a depth of about 18 to 20 km (ref.^16,66^). In this respect, Kopp *et al*.^67^ assigned density values to similar sedimentary units in the neighbouring Java trench of between 2.23 and 2.40 g cm^−^³, with an average of 2.32 g cm^−^³. The density of the oceanic crust was assumed to have a uniform value of 2.89 g cm^−^³, followed by the lithospheric mantle with a density of 3.37 g cm^−^³ (ref.^67^). These values are employed here for pressure-to-depth conversion of our thermobarometric results.

### Helium Isotope Geochemistry

Pyroxene crystals for isotope analysis (≥2 g) were hand-picked at Uppsala University from crushed aliquots of the 1963 Agung samples and subsequently cleaned in an ultrasonic bath using an acetone-methanol solvent mixture. A crystal aliquot of approximately 1 g was loaded into an online, electromagnetic crusher attached to the gas purification line of a noble gas spectrometer (MAP 215) at the Fluids and Volatiles Laboratory at Scripps Institution of Oceanography in La Jolla, California, USA. The device was pumped to ultrahigh vacuum overnight before the sample was crushed by a magnetised steel slug that was accelerated externally to a frequency of ~120 impacts per minute for a duration of 2 minutes (for full method description see Scarsi *et al*.^68^ and Shaw *et al*.^69^). During the crushing process, volatiles released from melt inclusions in the crystals were filtered through a combination of cooled charcoal traps and titanium and Zr-Al alloy getters in the mass spectrometer purification line. Prior to analysis for abundance and isotope ratios, He was separated from Ne. Standards used for analysis were SIO air (=1R_A_) or Murdering Mudpots He (=16.45 R_A_). The helium ratio is reported as ^3^He/^4^He and can be found in the Supplementary Information.

### Oxygen Isotope Geochemistry

Pyroxene, feldspar and olivine crystals were prepared by hand-picking inclusion-poor grains under a binocular microscope and subsequent cleaning in an ultrasonic bath. Laser fluorination of 2.5–3 mg crystal aliquots per independent run were carried out at the University of Cape Town, South Africa (for full analytical details, see Harris & Vogeli^70^). Resulting oxygen isotope ratios are reported in standard δ-notation relative to SMOW (Standard Mean Ocean Water), where δ = [(^18^O/^16^O)sample/(^18^O/^16^O)_SMOW_ − 1) ∗ 1000. The raw data were normalised and corrected for reference gas drift using the internal standard MON GT^70^ (Monastery garnet, δ^18^O = 5.38‰). The long-term average difference in δ^18^O values of duplicates of MON GT analysed during this study was 0.14‰ (n = 216), which corresponds to a 2σ value of 0.19‰.

### Data Availability

All data generated or analysed during this study are included in this published article (and its Supplementary Information files).

## Electronic supplementary material

Supplementary Information

Dataset 1
