# Supplementary material for: Multi-level magma plumbing at Agung and Batur volcanoes increases risk of hazardous eruptions
Source: Sci Rep. 2018 Jul 12;8:10547. doi: 10.1038/s41598-018-28125-2 (PMC6043508; doi:10.1038/s41598-018-28125-2)
Supplement: Supplementary file 1 — Supplementary Information [file 41598_2018_28125_MOESM1_ESM.docx]

**Multi-level magma plumbing at Agung and Batur volcanoes increases risk of hazardous eruptions**

Harri Geiger^1^, Valentin R. Troll^1,2*^, Ester M. Jolis^1,3^, Frances M. Deegan^1,2^, Chris Harris^4^, David R. Hilton^5^ and Carmela Freda^2^

**^1^**Section for Mineralogy, Petrology and Tectonics, Department of Earth Sciences, Uppsala University, Uppsala, Sweden

^2^Istituto Nazionale di Geofisica e Vulcanologia, Rome, Italy

^3^Geomar Helmholtz Centre for Ocean Research, Kiel, Germany

^4^Department of Geological Sciences, University of Cape Town, South Africa

^5^Geosciences Research Division, Scripps Institution of Oceanography, La Jolla, USA

*Corresponding author e-mail: valentin.troll@geo.uu.se

**Supplementary Information**

**Methods**

**Whole rock geochemistry**

Three lava samples (one each for the eruptions in 1963 of Agung, 1963 of Batur, and 1974 of Batur) were cut to remove weathered surfaces prior to jaw crushing and powdering in an agate mill at Uppsala University, Sweden. Major and trace element compositions of bulk rocks were analysed at Acme Analytical Laboratories, Vancouver, Canada ([http://acmelab.com](https://www.google.com/url?q=http://acmelab.com&sa=D&ust=1518523899170000&usg=AFQjCNHvf_GqO1mD-PLnvQsvvd9ARKK9mw)) using LiBO_2_/Li_2_B_4_O_7_ fusion ICP-ES analysis for major elements and acid digestion ICP-MS analysis for trace elements. Method detection limits (MDLs) for major elements as oxides are 0.01 wt% except for Fe_2_O_3_ (0.04 wt%) and Cr_2_O_3_ (0.002 wt%). For trace elements, MDLs are in the range of 0.002 ppm to 20 ppm. Sample duplicates have a reproducibility of < 0.10 wt% for most major elements and < 10 ppm for trace elements. Accuracy of the methods was confirmed by internal reference materials (SO-18 for major elements, OREAS-24P and OREAS-45P for trace elements), with a reproducibility of < 0.10 wt% for major elements and < 5 ppm for trace elements.

**Results**

**Clinopyroxene (-melt) thermobarometry**

Results from the K_D_(Fe-Mg) equilibrium test (**Putirka 2008**) applied to clinopyroxene from the 1963 eruption of Agung coupled with whole-rock and rasterized matrix compositions (data obtained in this study), as well as clinopyroxene together with literature data on matrix glass and clinopyroxene inclusions from **Self & King (1996**) are shown in figure **S1a**. Most data points fall within the K_D_(Fe-Mg) = 0.28 ± 0.08 equilibrium envelope when coupled with matrix glass and rasterized matrix analyses, whereas most pairs of clinopyroxene coupled with whole-rock or melt inclusions fall outside the equilibrium envelope and are not considered further. The second equilibrium test was performed by comparing the predicted versus observed clinopyroxene components for the two remaining melt compositions (CaTs, EnFs, DiHd, Jd). This test yielded 81% of all matrix glass data points (DiHd; **Fig.S1b**) and 37% of all rasterized matrix data points to fall within the ±0.10 envelope (DiHd; **Fig.S1c**), qualifying the matrix glass composition as the most suitable observed equilibrium melt.

For the 1963 eruption of Agung, applying the PT08Al formulation to the remaining equilibrium clinopyroxene and matrix glass pairs, crystallisation pressures of 364 to 905 MPa are obtained (**Fig. S1d**). These pressures translate to crystallisation depths between 11 and 27 km. Employing the PT08Jd model on the same set of equilibrium clinopyroxene-melt pairs yields a pressure range from 389 to 1128 MPa, which translates to crystallisation depths between 12 and 33 km (**Fig. S1d**). Pressures of 341 to 820 MPa are derived for clinopyroxene using the PT08Nim model (**Fig. S1d**) and these values correspond to crystallisation depths between 10 and 24 km, which is a more limited pressure-depth range that largely overlaps with the results from PT08Al and PT08Jd.

For the 1963 Batur eruption, clinopyroxene was coupled with whole-rock and raster-analysed matrix compositions (**de Hoog et al. 2001; Reubi & I. A. Nicholls 2004**; this study). Applying the K_D_(Fe-Mg) equilibrium test to clinopyroxene and the possible melt compositions defines the two whole-rock analyses (**de Hoog et al. 2001; Reubi and Nicholls 2004**) and the rasterized matrix composition as viable melts (**Fig. S2a**). The predicted vs. observed mineral component test resulted in the whole-rock analysis of **de Hoog et al. (2001)** and the rasterized matrix composition from this study as best nominal melts with all of the matched data points falling within the accepted equilibrium envelope (**Fig. S2b-d**). These two melt compositions were therefore utilised in the thermobarometric calculations.

When using PT08Al on clinopyroxene-matrix pairs from the 1963 Batur eruption, the resulting pressures range from 262 to 663 MPa, which translates to between 9 and 23 km depth (Fig. S2e). In turn, PT08Jd results in a pressure range for the equilibrium crystal-matrix pairs of 331 to 583 MPa, which corresponds to a crystallisation depth of 11 to 20 km (**Fig. S2e**). Using the PT08Al formulation on clinopyroxene and equilibrium whole-rock data from **de Hoog et al. (2001)** resulted in a pressure range from 151 to 542 MPa, which translates to a crystallisation depth of between 5 and 19 km (**Fig. S2f**). The PT08Jd model, in turn, calculates pressures for the crystal-whole rock pairings to lie between 320 and 576 MPa, or 11 and 20 km depth (**Fig. S2f**).

Three nominal melts were tested in combination with clinopyroxene from the 1974 Batur eruption. Besides whole-rock and the rasterized matrix compositions from this study, whole-rock data from **de Hoog et al. (2001**) were employed. The K_D_(Fe-Mg) equilibrium test defines the rasterized matrix composition as the best fit nominal melt (**Fig. S3a**). However, the observed vs. predicted mineral component test shows a better fit for the two whole-rock analyses, with 99% of the correlated data points falling within the equilibrium envelope (**Fig. S3b-d**).

Applying the PT08Al formulation on the remaining nominal melts in combination with clinopyroxene from the 1974 Batur lavas leads to pressure results that range from 302 to 637 MPa (10 to 22km depth) for the whole-rock composition from this study (**Fig. S3e**) and from 284 to 620 MPa (10 to 21 km depth) for the whole-rock composition from **de Hoog et al. (2001)** (**Fig. S3f**). The results from PT08Jd yield pressures of 352 to 670 MPa (12 to 23 km depth) for whole-rock from this study (**Fig. S3e**) and pressures from 357 to 675 MPa (12 to 23 km depth) for whole-rock data from **de Hoog et al. (2001)** (**Fig. S3f**). Similar to Agung, PT08Nim was used to verify the results from clinopyroxene-melt thermobarometry. For 1963 clinopyroxene, PT08Nim calculated pressures to lie between 277 and 498 MPa (11 to 20 km depth; **Fig. S3e**), whereas for clinopyroxene from the 1974 eruption, pressures between 239 and 615 MPa (8 to 21 km depth; **Fig. S3f**) were obtained by this approach.

**Plagioclase-melt thermobarometry**

Additional data sets were considered as possible nominal melts for plagioclase-melt thermobarometry, including whole-rock and rasterized matrix data acquired in this study, and matrix glass and plagioclase inclusions from **Self & King (1996)**. Results from the K_D_(Ab-An) equilibrium test after **Putirka (2008)** are shown in figure **S4a**. Data points were evaluated on the basis of their calculated K_D_ values and temperature estimates, which resulted in two possible equilibrium envelopes of 0.10 ± 0.05 for T ≤ 1050 °C and 0.27 ± 0.11 for T ≥ 1050 °C. Calculated temperatures were < 1050°C for any given nominal melt, therefore only the K_D_ = 0 .10 ± 0.05 envelope has been applied to the 1963 Agung lavas (Fig. S4a). The most suitable nominal melt is the rasterized matrix composition from this study with ~30% of the evaluated mineral-melt pairs satisfying the defined equilibrium conditions. The plagioclase-melt thermobarometry formulation of **Putirka (2008)** yields crystallisation pressures between 131 and 200 MPa for the 1963 eruption of Agung, which correspond to a depth range of ~4.5 to 7 km (**Fig. S4b**).

Plagioclase from the 1963 Batur eruption was coupled with whole-rock and rasterized matrix composition from this study as well as with whole-rock data from **de Hoog et al. (2001)** and **Wheller & Varne (1986)**. The resulting mineral-melt couples span both K_D_(Ab-An) equilibrium temperature ranges, however, chemical equilibrium is only exhibited by mineral-melt couples falling into the K_D_ = 0.10 ± 0.05 envelope. The equilibrium test shows, moreover, that the rasterized matrix composition is the most suitable nominal melt with 68% of data points passing the test (**Fig. S4c**). Resulting pressures for equilibrium mineral-melt pairs from the 1963 Batur eruption range from 79 to 147 MPa, which corresponds to a crystallisation depth of ~3 to 5 km (**Fig. S4d**).

For the 1974 Batur lavas, plagioclase was tested against whole-rock and rasterized matrix data from this study as well as against whole-rock data from **de Hoog et al. (2001)** and **Wheller & Varne (1986)**. Similar to plagioclase in the 1963 Batur lavas, temperatures obtained from thermobarometric calculations span both K_D_(Ab-An) temperature ranges. Again, only mineral-melt couples that fall into the K_D_ = 0.10 ± 0.05 envelope satisfy equilibrium conditions, and whole-rock compositions from this study were found to be the most suitable nominal melt with 32% of data points displaying chemical equilibrium (**Fig. S4e**). Thermobarometry on equilibrium mineral-melt couples from the 1974 Batur eruption resulted in pressures between 104 and 235 MPa, which translates to a depth of ~4 to 8 km (**Fig. S4f**).


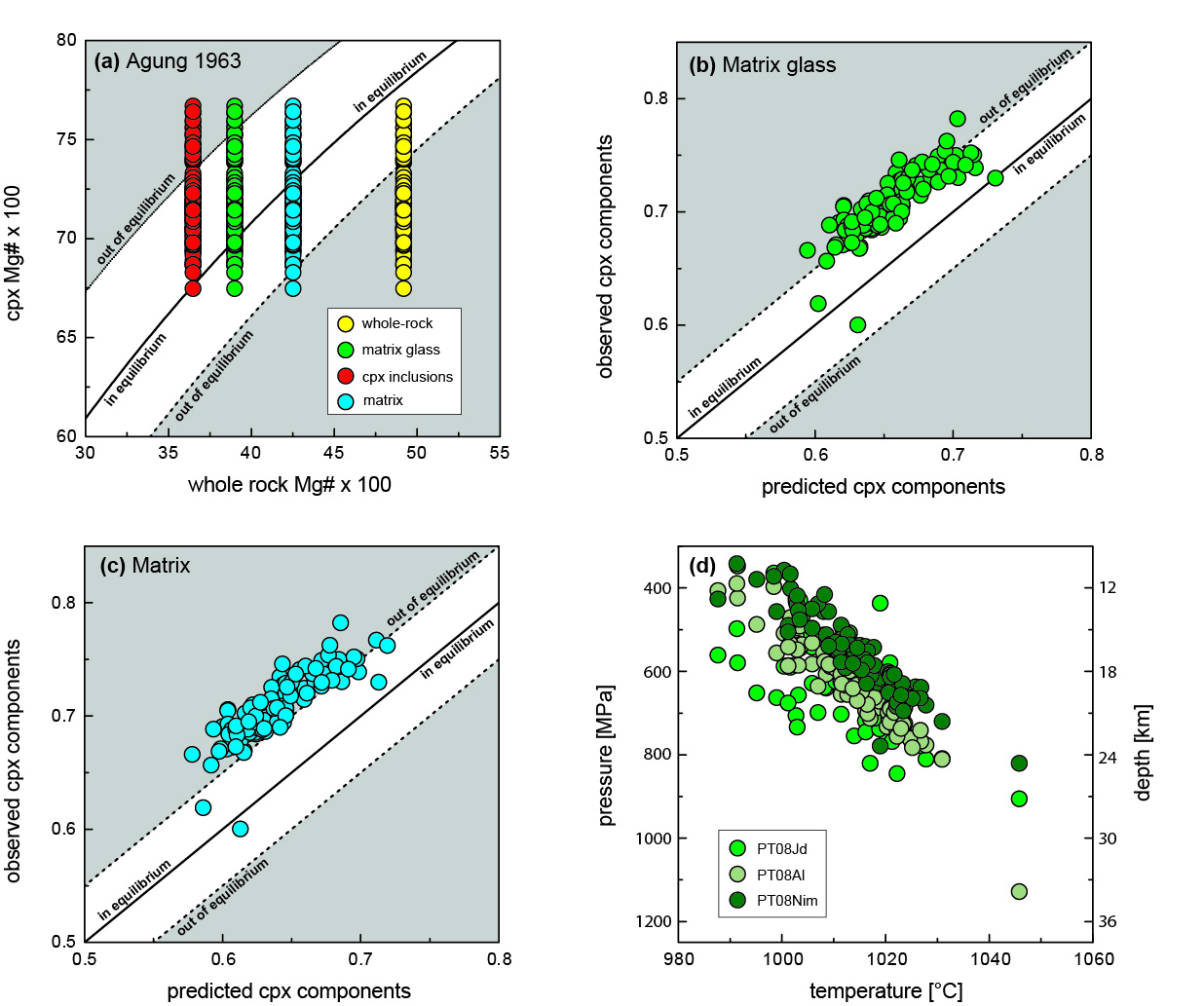


**Figure S1: a)** The K_D_(Fe-Mg) equilibrium test after **Putirka (2008)** applied to clinopyroxene from the 1963 eruption of Agung coupled with possible nominal melts. The predicted vs. observed (diopside-hedenbergite) clinopyroxene component equilibrium test for matrix composition **(b)** and matrix glass **(c)**. Results from clinopyroxene-melt thermobarometry and clinopyroxene composition barometry (after **Putirka 2008**) for selected nominal melts for the 1963 Agung eruption **(d)**. Pyroxene from Agung yield a main crystallisation pressure interval of between 400 and 800 MPa.


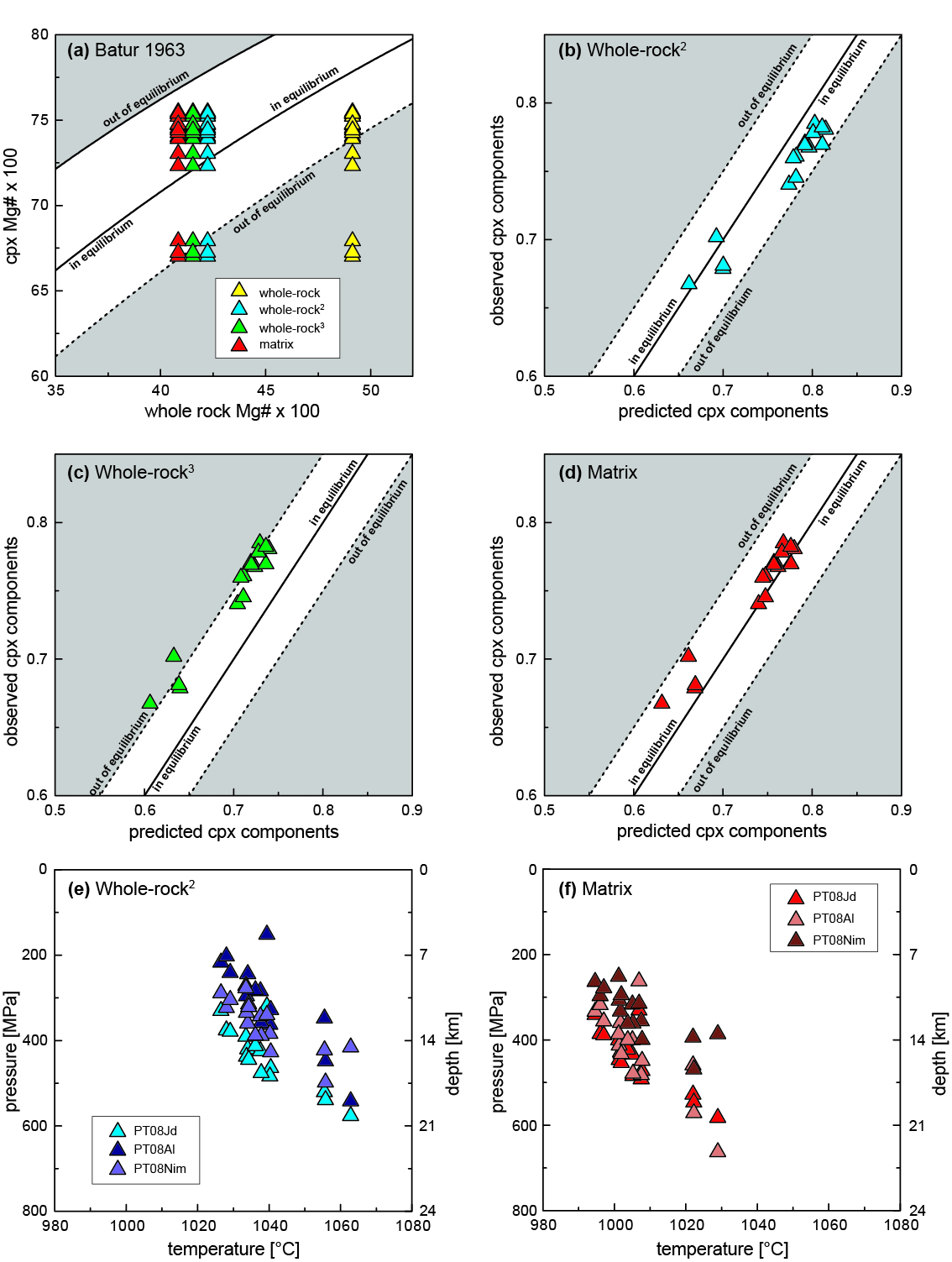


**Figure S2: a)** The K_D_(Fe-Mg) equilibrium test after **Putirka (2008)** applied to clinopyroxene from the 1963 eruption of Batur coupled with a range of possible nominal melts. The predicted vs. observed (diopside/hedenbergite) clinopyroxene component equilibrium test for **b)** whole-rock from **de Hoog et al. (2001)**, **c)** whole-rock from **Reubi & Nicholls (2004)** and **d)** matrix composition (this study) as possible nominal melts. Results from clinopyroxene-melt thermobarometry and clinopyroxene composition barometry (**Putirka 2008**) for 1963 Batur clinopyroxene coupled with **e)** whole-rock and **f)** matrix compositions as possible nominal melts. Pyroxene crystallisation pressures cluster dominantly between 200 and 600 MPa for both possible nominal melts.

**
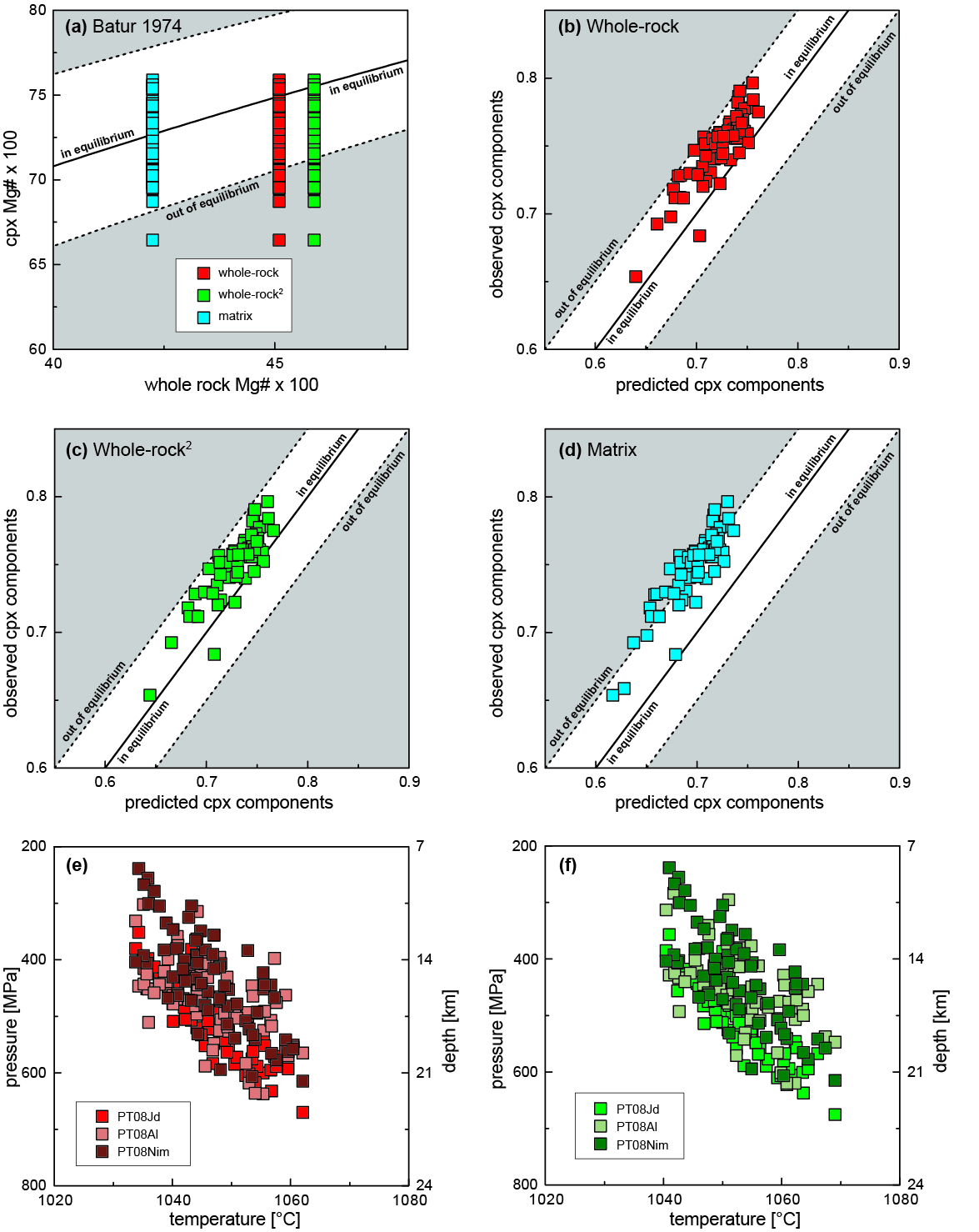
**

**Figure S3: a)** The K_D_(Fe-Mg) equilibrium test after **Putirka (2008)** applied to clinopyroxene from the 1974 eruption of Batur coupled with various possible nominal melts. The predicted vs. observed (diopside-hedenbergite) clinopyroxene component equilibrium test for **b)** whole-rock of this study, **c)** whole-rock from **de Hoog et al. (2001)**, and **d)** matrix composition as possible nominal melts. Results from clinopyroxene-melt thermobarometry and clinopyroxene composition barometry (**Putirka 2008**) for 1974 Batur clinopyroxene coupled with **e)** whole-rock (this study) and **f)** whole-rock from **de Hoog et al. (2001)**. Clinopyroxene from the 1974 Batur eruption records crystallisation pressures that cluster dominantly around 200-620 MPa.


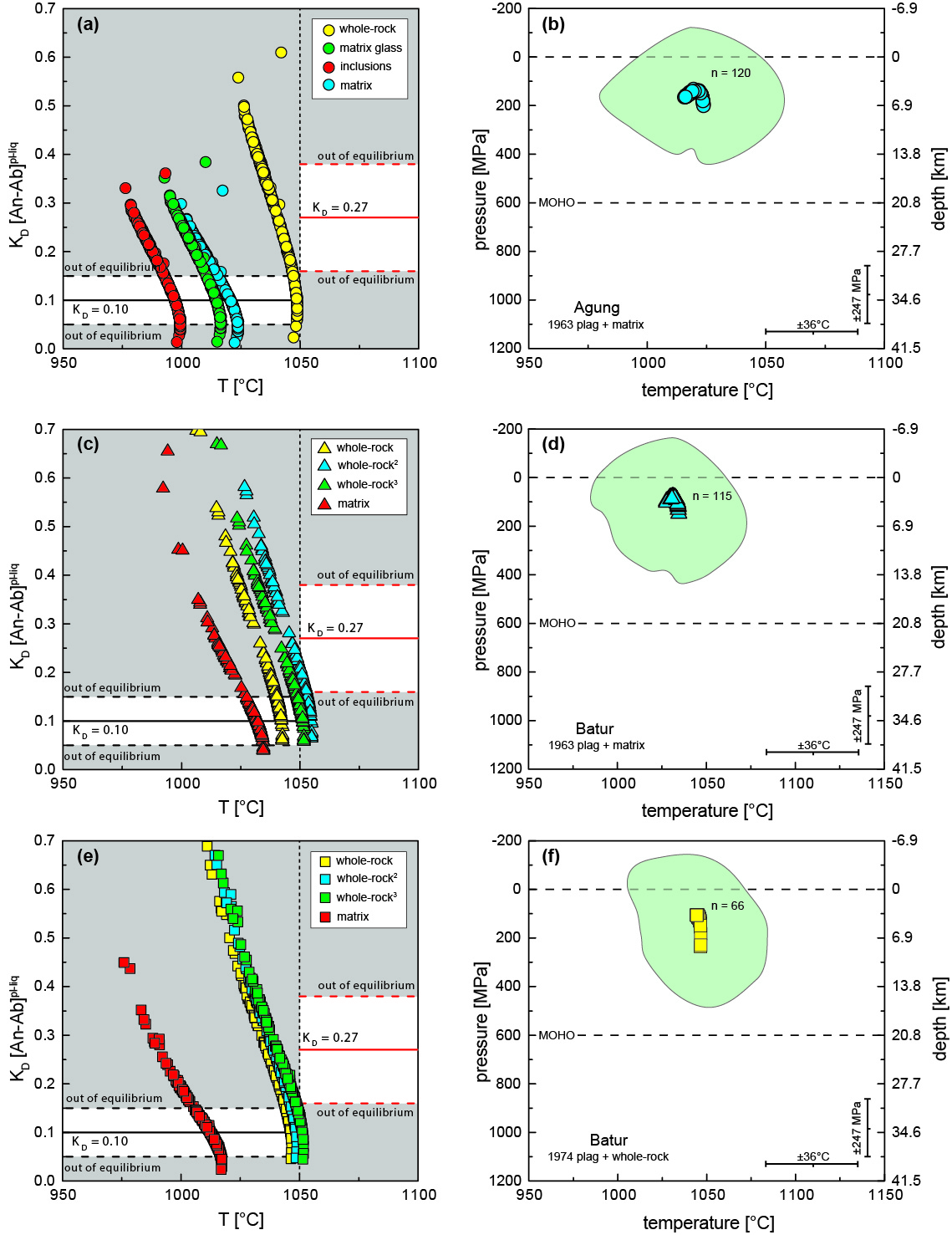


**Figure S4:** The K_D_(Ab-An) equilibrium test after **Putirka (2008)** applied to plagioclase coupled with a range of possible nominal melt compositions for **a)** the 1963 Agung eruption, **c)** the 1963 Batur eruption, and **e)** the 1974 Batur eruption. Results for plagioclase-melt thermobarometry for plagioclase coupled with matrix composition from the 1963 eruptions of Agung and Batur, and whole-rock composition from the 1974 Batur eruption are shown in **b)**, **d)**, and **f)**, respectively. Error clouds (SEE) are marked in light green. Agung plagioclase crystallised dominantly at 100 to 300 MPa, whereas plagioclase from Batur show crystallisation pressures of 50 to 200 MPa for the 1963 eruption, and between 100 and 300 MPa for the 1974 eruption.

| **Table S1:** **Helium isotope data for Agung** | | | | | | | | | |
| --- | --- | --- | --- | --- | --- | --- | --- | --- | --- |
| **Sample ID** | **Sample wt. (g)** | **Mineral** | **%blank on ^4^He** | **^3^He/^4^He (R/R_A_)raw*** | **^3^He/^4^He (R/R_A_)bc^#^** | **X^&^** | **^3^He/^4^He (R_c_/R_A_)^¤^** | **±σ** | **[^4^He]c (x10^-9^) cm^3^ STP/g^†^** |
| A-BA1-1963 | 1.2528 | pyroxene | 4.5 | 6.96 | 7.42 | 6.3 | 8.62 | 0.39 | 0.78 |

Table S1: Helium isotope data in pyroxene from the 1963 Agung eruption. Indices description: *. measured ratios – #. blank corrected ratios – &. X = (^4^He/^20^Ne)M/(^4^He/^20^Ne)air, M is the measured ^4^He/^20^Ne ratio – ¤. air-corrected He isotope ratio = [(R/R_A_*X)-1]/(X-1) – †. Helium abundance data are corrected for air contamination where [He]_C_ = ([He]M×(X−1))/X. Uncertainty ± 5%.

| **Table S2: Oxygen isotope data for Agung (A-BA1) and Batur (B-BA2)** | | | |
| --- | --- | --- | --- |
| **Sample ID** | **Sample type** | **δ^18^O‰ [measured]** | **δ^18^O‰**  **[calculated melt]** |
| B-BA2-63 ol - 1 | Olivine | 4.8 | 5.2 |
| B-BA2-63 ol - 2 | Olivine | 4.9 | 5.3 |
| B-BA2-63 cpx | Clinopyroxene | 5.2 | 5.5 |
| B-BA2-74 cpx - 1 | Clinopyroxene | 5.0 | 5.3 |
| B-BA2-74 cpx - 2 | Clinopyroxene | 5.6 | 5.9 |
| A-BA1-63 cpx - 1 | Clinopyroxene | 5.4 | 5.7 |
| A-BA1-63 cpx - 2 | Clinopyroxene | 5.3 | 5.6 |
| A-BA1-63 cpx - 3 | Clinopyroxene | 5.8 | 6.1 |
| B-BA2-63 plag - 1 | Plagioclase | 5.7 | 5.5 |
| B-BA2-63 plag - 2 | Plagioclase | 5.9 | 5.7 |
| B-BA2-74 plag - 1 | Plagioclase | 5.5 | 5.3 |
| B-BA2-74 plag - 2 | Plagioclase | 6.0 | 5.8 |
| A-BA1-63 plag - 1 | Plagioclase | 6.0 | 5.8 |
| A-BA1-63 plag - 2 | Plagioclase | 6.4 | 6.2 |

Table S2: Oxygen isotope data obtained by laser fluorination for olivine, clinopyroxene, and plagioclase mineral separates (where available) in lavas from the 1963 Agung and the 1963/74 Batur eruptions. The raw data were normalised and corrected for reference gas drift using the internal standard MON GT (Monastery garnet, δ^18^O = 5.38‰, Harris & Vogeli 2010). The long-term average difference in δ^18^O values of duplicates of MON GT analysed during this study was 0.14‰ (n = 216), which corresponds to a 2σ value of 0.19‰. To estimate the magma δ^18^O values from the mineral data we assume δ^18^O fractionation of –0.2‰ for plagioclase, +0.3‰ for pyroxene (Harris et al. 2005), and +0.4‰ for olivine (Eiler et al. 2000).

| **Table S3: References for regional comparison** | |
| --- | --- |
| **Volcano** | **References** |
| Anak-Krakatau | **(Jaxybulatov et al. 2011; Dahren et al. 2012)** |
| Gede | **(Handley et al. 2010)** |
| Merapi | **(Chadwick et al. 2013; Costa et al. 2013; Nadeau et al. 2013; Preece et al. 2014; Deegan et al. 2016)** |
| Kelut | **(Jeffery et al. 2013)** |

Table S3: References for “Magma storage along the Java-Bali segment of the Sunda arc”.

**References**

Chadwick JP, Troll VR, Waight TE, et al (2013) Petrology and geochemistry of igneous inclusions in recent Merapi deposits: a window into the sub-volcanic plumbing system. Contrib to Mineral Petrol 165:259–282 . doi: 10.1007/s00410-012-0808-7

Costa F, Andreastuti S, Bouvet de Maisonneuve C, Pallister JS (2013) Petrological insights into the storage conditions, and magmatic processes that yielded the centennial 2010 Merapi explosive eruption. J Volcanol Geotherm Res 261:209–235 . doi: 10.1016/j.jvolgeores.2012.12.025

Dahren B, Troll VR, Andersson UB, et al (2012) Magma plumbing beneath Anak Krakatau volcano, Indonesia: evidence for multiple magma storage regions. Contrib to Mineral Petrol 163:631–651 . doi: 10.1007/s00410-011-0690-8

de Hoog JCM, Taylor BE, van Bergen MJ (2001) Sulfur isotope systematics of basaltic lavas from Indonesia : implications for the sulfur cycle in subduction zones. Earth Planet Sci Lett 189:237–252

Deegan FM, Whitehouse MJ, Troll VR, et al (2016) Pyroxene standards for SIMS oxygen isotope analysis and their application to Merapi volcano, Sunda arc, Indonesia. Chem Geol 447:1–10 . doi: 10.1016/j.chemgeo.2016.10.018

Eiler JM, Crawford A, Elliott T, et al (2000) Oxygen Isotope Geochemistry of Oceanic-Arc Lavas. J Petrol 41:229–256 . doi: 10.1093/petrology/41.2.229

Handley HK, Macpherson CG, Davidson JP (2010) Geochemical and Sr–O isotopic constraints on magmatic differentiation at Gede Volcanic Complex, West Java, Indonesia. Contrib to Mineral Petrol 159:885–908 . doi: 10.1007/s00410-009-0460-z

Harris C, Pronost JJM, Ashwal LD, Cawthorn RG (2005) Oxygen and hydrogen isotope stratigraphy of the Rustenburg Layered Suite, Bushveld Complex: Constraints on crustal contamination. J Petrol 46:579–601 . doi: 10.1093/petrology/egh089

Harris C, Vogeli J (2010) Oxygen isotope composition of garnet in the Peninsula Granite, Cape Granite Suite, South Africa: Constraints on melting and emplacement mechanisms. South African J Geol 113:401–412 . doi: 10.2113/gssajg.113.4.401

Jaxybulatov K, Koulakov I, Seht MI, et al (2011) Evidence for high fluid/melt content beneath Krakatau volcano (Indonesia) from local earthquake tomography. J Volcanol Geotherm Res 206:96–105 . doi: 10.1016/j.jvolgeores.2011.06.009

Jeffery AJ, Gertisser R, Troll VR, et al (2013) The pre-eruptive magma plumbing system of the 2007-2008 dome-forming eruption of Kelut volcano, East Java, Indonesia. Contrib to Mineral Petrol 166:275–308 . doi: 10.1007/s00410-013-0875-4

Nadeau O, Williams-Jones AE, Stix J (2013) Magmatic–hydrothermal evolution and devolatilization beneath Merapi volcano, Indonesia. J Volcanol Geotherm Res 261:50–68 . doi: 10.1016/j.jvolgeores.2013.04.006

Preece K, Gertisser R, Barclay J, et al (2014) Pre- and syn-eruptive degassing and crystallisation processes of the 2010 and 2006 eruptions of Merapi volcano, Indonesia. Contrib to Mineral Petrol 168:1061 . doi: 10.1007/s00410-014-1061-z

Putirka KD (2008) Thermometers and Barometers for Volcanic Systems. Rev Mineral Geochemistry 69:61–120 . doi: 10.2138/rmg.2008.69.3

Reubi O, Nicholls IA (2004) Variability in eruptive dynamics associated with caldera collapse: an example from two successive eruptions at Batur volcanic field, Bali, Indonesia. Bull Volcanol 66:134–148 . doi: 10.1007/s00445-003-0298-6

Self S, King AJ (1996) Petrology and sulfur and chlorine emissions of the 1963 eruption of Gunung Agung, Bali, Indonesia. Bull Volcanol 58:263–285 . doi: 10.1007/s004450050139

Wheller GE, Varne R (1986) Genesis of dacitic magmatism at Batur volcano, Bali, Indonesia: Implications for the origins of stratovolcano calderas. J Volcanol Geotherm Res 28:363–378
